# Biomechanical changes in females with poly cystic ovarian syndrome: a case–control study

**DOI:** 10.1038/s41598-025-93481-9

**Published:** 2025-04-01

**Authors:** Eman E. Kamal, Hamada A. Hamada, Reda Sayed Ashour, Amel M. Yousef, Rovan M. Elbesh

**Affiliations:** 1 Department of Physical Therapy for Women’s Health, Faculty of Physical Therapy, Al Ryada University for Science and Technology, Sadat City, Egypt; 2 Department of Biomechanics, Faculty of Physical Therapy, Cairo University, Cairo, Egypt; 3 Department of Physical Therapy for Women’s Health, Faculty of Physical Therapy, Cairo University, Cairo, Egypt; 4 Department of Physical Therapy for Women’s Health, Faculty of Physical Therapy, Misr University for Science and Technology, Giza, Egypt

**Keywords:** Biomechanical changes, Spinal alignment, Polycystic ovarian syndrome, Hormonal imbalance, Low-grade inflammation, Bone, Orthopaedics, Signs and symptoms, Endocrinology, Reproductive disorders, Health care, Physical examination

## Abstract

Polycystic ovarian syndrome (PCOS) is a prevalent endocrine disorder that causes an inversion of the normal luteinizing hormone (LH) to follicle-stimulating hormone (FSH) ratio. Females with PCOS also experience chronic inflammation. This hormonal imbalance and persistent inflammation can reduce muscle strength and mass. Consequently, this may affect the lumbopelvic muscles, potentially leading to postural abnormalities and spinal misalignment. The study’s goals were to find out how the biomechanics of women with PCOS differ from those who did not have the condition and to confirm the link between lumbopelvic parameters and the LH/FSH ratio in women with PCOS. The researcher conducted a case–control study on 95 nulliparous females, with 52 having PCOS and classified as a study group and 43 as a control group. The participants ranged in age from 25 to 35 years, and their body mass index ranged from 25 to 29.9 kg/m^2^. All participants were selected from the gynecological outpatient clinic of Om El-Masryeen Hospital. The researcher used a pelvic inclinometer to evaluate the pelvic inclination angle and an inclinometer to examine the lumbar angle. Additionally, the researcher simultaneously collected blood samples on the third day of the menstrual cycle. Females with PCOS had significantly higher pelvic inclination and lumbar curve angles than controls (*p* < 0.05). LH/FSH ratio strongly correlated with lumbar angle and pelvic inclination. Females with PCOS had greater pelvic tilting and exaggerated lumbar lordosis than controls. The LH/FSH ratio showed a strong correlation with both the lumbar curve angle and pelvic inclination in PCOS.

Clinical trial: The clinical trial number [NCT03740932] with initial release date at 09/17/2024.

## Introduction

PCOS, the most common endocrine condition among women of reproductive age, is a multifactorial disorder with various genetic, metabolic, endocrine, and environmental abnormalities^1^. It is characterized by reproductive and metabolic disturbances, and the presence of at least two of the following three criteria: hyperandrogenism (biochemical or clinical), ovulatory dysfunction (Olig ovulation or anovulation), and polycystic ovary morphology^1^. Globally, PCOS affects 10–15% of women, burdening many countries’ economies with its direct and indirect consequences^2^.

Increasing evidence suggests that PCOS has lifelong effects on women, can begin in utero in those with a genetic risk, manifests clinically at puberty, and continues during the reproductive years^3^. Despite having a high incidence, PCOS is a very heterogeneous disorder, which may have contributed to the fact that its underlying cause is still unknown^3^. Functional ovarian hyperandrogenism is the known cause of PCOS. Most females with PCOS (about two-thirds) have functional ovarian hyperandrogenism. This is a strange reaction that happens when luteinizing hormone (LH) causes the overproduction of androgens. Excess insulin levels may be the cause of this hormonal imbalance, as they increase the ovaries’ sensitivity to LH^4^.

Ovarian hyperandrogenism in PCOS is associated with a disruption of the normal pulsatile release of gonadotropin-releasing hormone. This disruption produces significant increase in the level of luteinizing hormone (LH) compared to follicle-stimulating hormone (FSH). This hormonal shift stimulates androgen production in the ovaries while suppressing estrogen synthesis, creating a vicious cycle that perpetuates the imbalance^5^. PCOS is a complex condition characterized by a cyclical interplay of insulin resistance, disturbed folliculogenesis, and abnormal gonadotropin dynamics^6^. Another significant factor in the pathophysiology of PCOS is the inflammation that is present in PCOS patients, irrespective of their adiposity or BMI^6^.

Persistent low-grade inflammation is a key factor associated with other PCOS characteristics such as insulin resistance, cardiovascular disease risk, and pelvic floor dysfunction in females^7^. Importantly, inflammation is also a hallmark of sarcopenia, a condition involving muscle loss and weakness. This connection suggests that the chronic inflammatory state in PCOS may contribute to muscle decline^8^.

Hormonal fluctuations can impact the pelvic floor muscles by altering their tension and length, affecting the pelvis, lower back, and hips^9^. The pathophysiology of pelvic floor dysfunction implicates hormonal imbalance, chronic low-grade inflammation, and reduced skeletal muscle strength and mass in females with PCOS. However, hormones play a crucial role in maintaining normal pelvic floor function throughout a woman’s life^10^. Saei et al. identified a significant correlation between elevated luteinizing hormone levels and pelvic floor dysfunction in females with PCOS^11^. The coordinated action of the pelvic floor and core muscles is essential for movement, balance, stability, and flexibility^9^. Weakened pelvic floor muscles can disrupt this core synergy, leading to impaired muscle coordination^12^. The relationship between spinal curvature and pelvic structure is complex^13^. Theoretically, the natural curves of the spine aid in distributing downward pressure on the pelvis^13^. However, abnormal spinal curvature may disrupt this balance, potentially contributing to pelvic floor dysfunction^13^.

Understanding the connection between posture and PCOS could revolutionize treatment approaches for women with this condition. The previously conducted studies revealed that PCOS is associated with impaired physical function in the form reduced core muscle endurance^14^, muscle and bone loss (osteosarcopenia)^15^, hand and knee osteoarthritis^16^ and increased LH/FSH ratio^15^. To the best of the authors’ knowledge, no study was conducted to discuss the biomechanical changes in patients with PCOS. Therefore, this study aimed to identify specific biomechanical changes in women with PCOS, focusing on how these changes impact the spine and pelvis and to verify the association between pelvic inclination, lumbar lordosis, and LH/FSH ratio in such cases. The findings could provide valuable insights for physical therapists and healthcare providers specializing in women’s health and musculoskeletal disorders.

## Materials and methods

### Study design

This was an observational case–control study.

### Participants

**Fig. 1:**
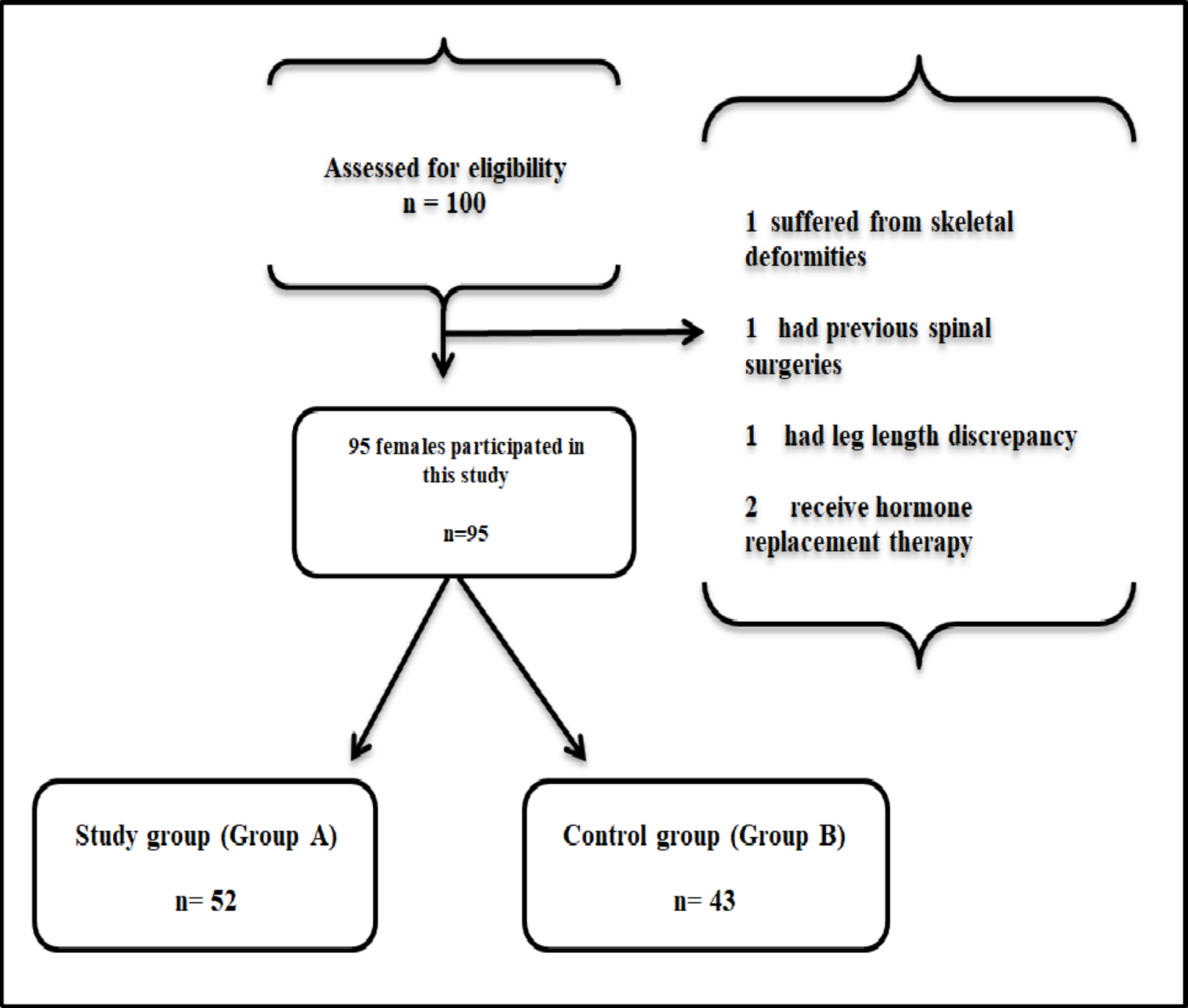
Flow chart indicating recruitment, randomization, and follow-up of the participants.

Ninety-five nulliparous females with no obstetric history participated in this study. All participants ranged from 25 to 35 years, with their body mass index (BMI) ranging from 25 to 29.9 kg/m^2^. Fifty-two females with PCOS and LH/FSH ratio greater than 1.5 were assigned to the study group (A). The gynecologist referred patients who met the Rotterdam PCOS Diagnostic Criteria for Adults^17^ which define PCOS as the presence of two of the following three criteria: oligo-anovulation (defined as less than eight menstrual cycles per year or more than 35 days between cycles), clinical/biochemical hyperandrogenism and polycystic ovarian morphology. The gynecologist referred 43 healthy females, who were not suffering from any gynecological condition to the control group (B). Participants who were obese with BMI greater than 30 kg/m^2^ were not allowed to take part in the study as obesity induces an increase in the anterior pelvic tilt and sacral inclination^18^. Participants were also excluded from the study if they had musculoskeletal deformities, previous gynecological or spinal surgeries, spinal deformities, leg length discrepancies, orthopedic or neurological disorders, or were on hormone replacement therapy^19^. This is because hormone replacement therapy changes the functional biomechanical properties by making the uterosacral ligament stiffer and the round ligament less stiff^20^ (Fig. 1).

### Procedures

**Fig. 2:**
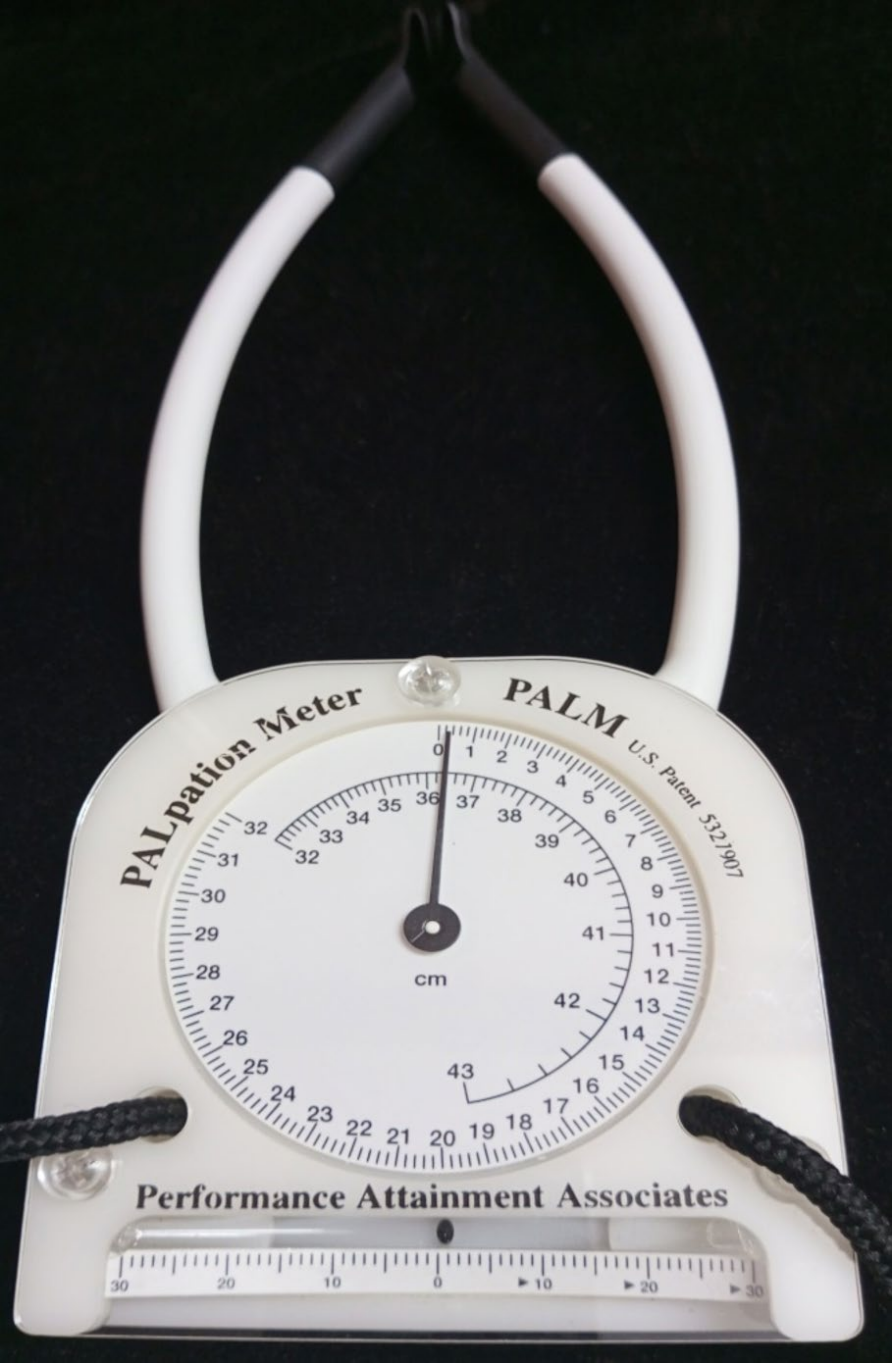
The pelvic inclinometer (PALM) with an inclinometer and two caliper arms.

**Fig. 3:**
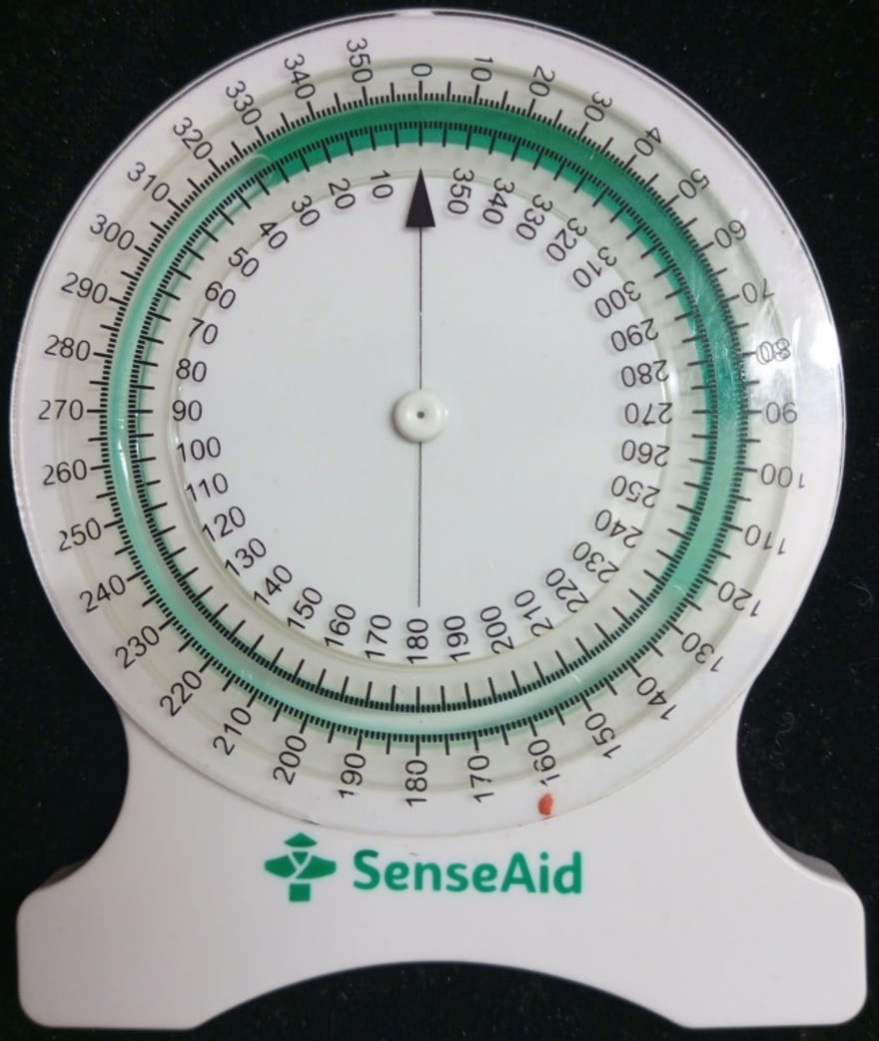
The bubble inclinometer, a rounded plastic inclinometer with a 360̊ rotating dial and fluid indicator.

The selected parameters for assessment were the pelvic sagittal inclination angle and the lumbar angle. The pelvic sagittal inclination angle was measured using the PALM inclinometer, which has an inclinometer and two calliper arms (Fig. 2). The PALM inclinometer is a semi-circular arc with a one-degree gradation ranging from 0° to 30° on either side of the midline. The design of the calliper arm tips ensures direct contact with the bony landmarks. A mounted protractor (calliper dial) with a 2 mm gradation ranging from 0 to 43 cm can measure the distance between the calliper arms (Performance Attainment Associates, US Patent 5327907). The lumbar angle was measured using the No-Leak PT Inclinometer for Range of Motion (ROM) Measurements by Sense Aid, White Plains, New York, USA. It is a round plastic inclinometer with a dial that can turn 360 degrees and a fluid indicator (Fig. 3).

Each angle was measured three times, and the mean was calculated to represent the data used for statistical analysis. All measurements were conducted in a relaxed, comfortable standing position, with the subject wearing loose clothing, bare feet, and positioned one foot apart on a level floor in the same room of the same building, at the same time of day. There was a fixed point in the wall, and the participant was instructed to look at it during the assessment; this point was adjusted at the level of the participant’s eyes. The females received no specific instructions on posture, allowing for measurements in a typical standing position. Data was recorded on a data collection sheet.

### Outcome measures

#### Pelvic tilt angle

**Fig. 4:**
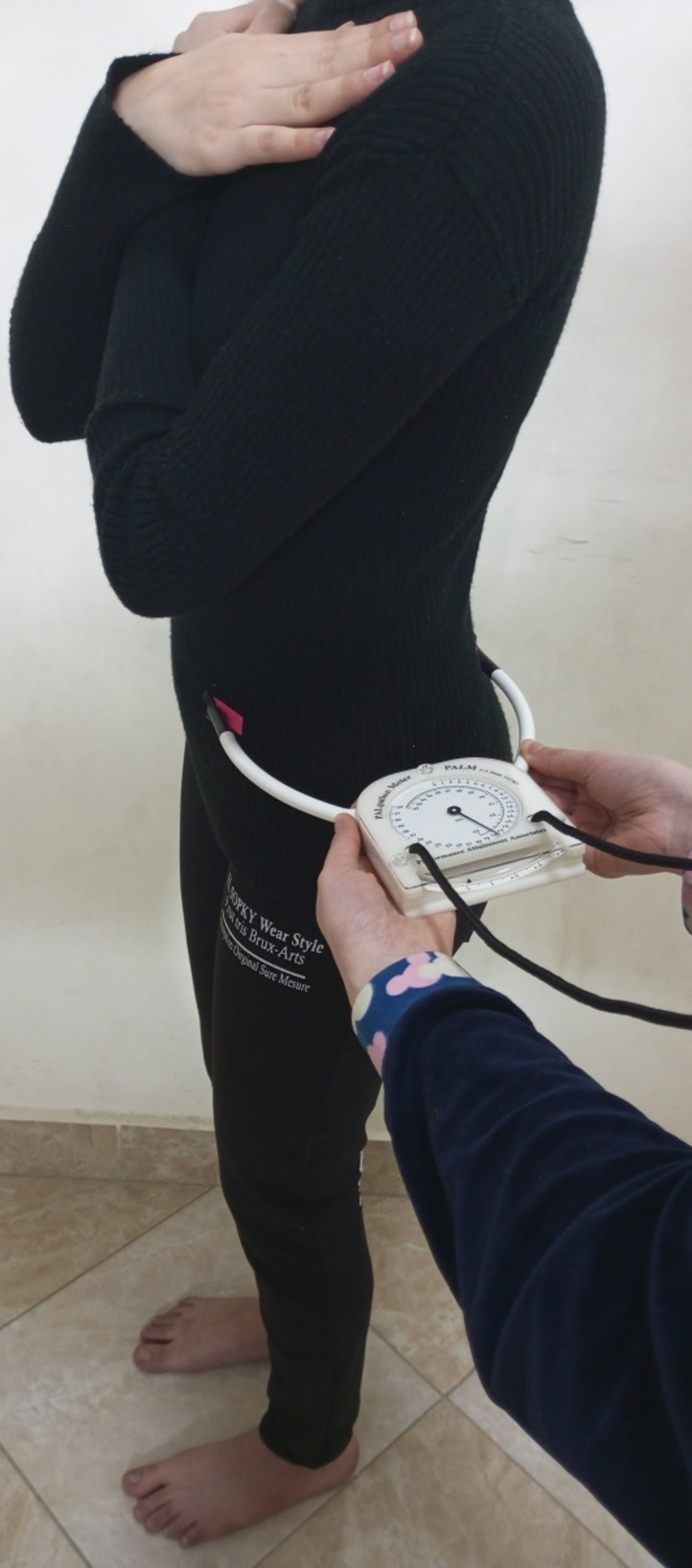
Measurement of the pelvic tilt angle while the patient was in upright standing position and the two caliper arms of the PALM inclinometer were placed on the patient’s anterior and posterior iliac spines.

While the participant was upright, the physiotherapist identified and marked the accurate locations of the anterior superior iliac spine (ASIS) and posterior superior iliac spine (PSIS). Then the therapist adjusted the PALM inclinometer’s one end arm on ASIS and the other on PSIS (Fig. 4). The PALM inclinometer’s bubble level showed that the pelvic sagittal inclination angle was the angle between a straight line and a line that went through the ASIS and PSIS. We took the previous measurement on both the right and left sides of the pelvis. This approach demonstrates solid intra-tester reliability (ICC = 0.87) as well as accuracy and validity^21^. The mean pelvic tilt angle value was reported to be 13° ± 6°^22^.

#### Lumbar angle

**Fig. 5:**
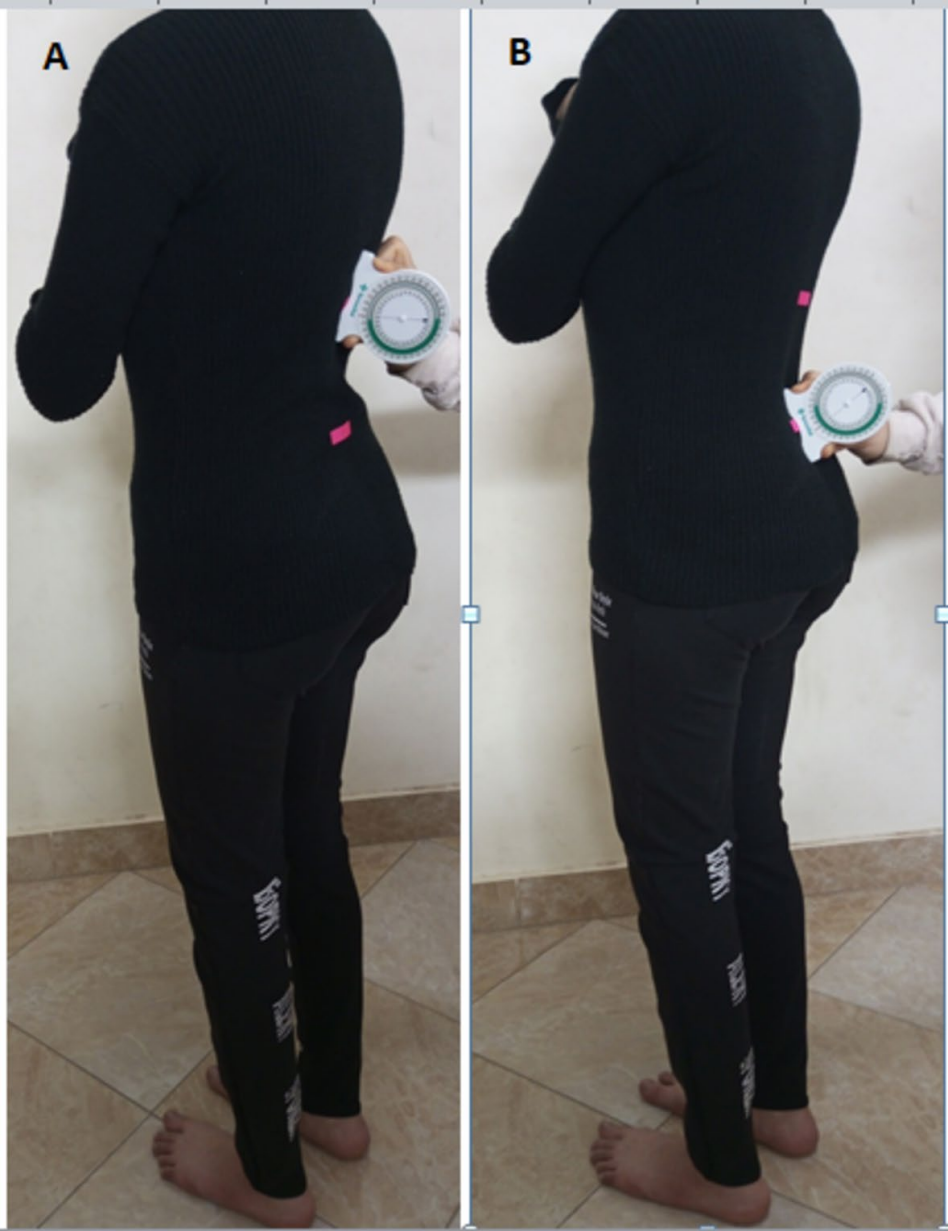
Measurement of the lumbar curve angle; (**A**) the dial of the inclinometer was set at 0 degree in T_12_/L_1_, (**B**) the dial reading at L_5_/S_1_ known from the inclinometer’s fluid level was recorded as the lumbar angle.

Before the examination, the physiotherapist identified the spinous processes of T_12_/L_1_/L_5_ and S_1_ by palpation and marked the inter-spinal spaces between T_12_/L_1_ and L_5_/S_1_ using adhesive stickers. Following that, the inclinometer was inserted gently but firmly into the marked inter-spinal spaces, thereby obtaining the lumbar angle. The dial of the inclinometer was set at 0 degree in T_12_/L_1_ (Fig. 5A) and the dial reading at L_5_/S_1_ (known from the inclinometer’s fluid level) was recorded (Fig. 5B). Neutral values of the lumbar angle are 20°–40°^23^. The researcher used the baseline inclinometer, which demonstrated high intra-rater reliability (ICC = 0.92) compared to the bubble inclinometer^24^.

#### LH and FSH measurements

##### Venipuncture timing

On the third day of the cycle, blood samples were drawn while fasting.

##### Measuring method

A laboratory examination of a sample of blood was used to measure the levels of FSH and LH.

##### Procedure

Blood sample was obtained from each patient in the morning (after overnight fasting) during the early follicular phase (day 3) of menstrual cycle. It was taken from a vein in the participant’s arm while they were in long sitting position after a 30-min resting period. First, the chemist disinfected the area with an alcohol wipe and tied an elastic band around participant’s upper arm to help expose the vein. Next, the needle was inserted and the blood was collected into the test tube. Once 5 ml blood was taken, the band was removed, and the needle was pulled out and the blood draw area was covered with gauze and a bandage.

### Data analysis

The researcher conducted the statistical analysis using SPSS for Windows, version 26 (SPSS, Inc., Chicago, IL). According to Levene’s test of homogeneity of variances, the variances and covariances were homogeneous (*p* > 0.05). Accordingly, parametric statistics were used. Quantitative variables were presented as mean ± standard deviation (X ± SD). The researcher used an unpaired *t*-test to compare the physical characteristics of both groups. A one-way between-subject MANOVA was conducted to determine the differences between both groups in the degree of pelvic inclination, lumbar curve angle, and hormones (LH and FSH). Pearson’s correlation coefficient was used to investigate the relationship between pelvic inclination, lumbar curve angle, and hormones (LH and FSH) in females with PCOS. The alpha level was set at 0.05, and the correlation coefficients were interpreted as 0–0.1 = extremely low, 0.10–0.30 = low, 0.30–0.50 = moderate, 0.50–0.70 = high, 0.70–0.90 = very high, and 0.90–1.0 = strong.

## Results

The statistical analysis of the physical characteristics (Table 1) revealed that there were no significant differences between both groups (*p* > 0.05). The one-way between-subject MANOVA revealed significant differences between both groups (F = 100.403, *p* < 0.01). Additionally, the pairwise tests showed significant increases in the mean values of all measured variables in group (A) compared with group (B) (*p* < 0.01). The conducted statistics were shown in Tables 2 and 3.

**Table 1:** Physical characteristics of participants in the study.

Physical characteristics	Study group (group A)		Control group (group B)		Unpaired t-test *P*-value
	Mean	± SD	Mean	± SD	
Age (years)	27.096	2.0123	24.697	2.262	0.303
BMI (kg/m^2^)	26.322	1.733	26.563	1.703	0.881

*Significant at alpha level < 0.05, *SD* standard deviation.

**Table 2:** The mean and standard deviation values of the measured variables in the study and control groups.

Measured variables	Study group (group A)		Control group (group B)	
	Mean	± SD	Mean	± SD
Right pelvic inclination	11.742	1.756	6.825	1.091
Left pelvic inclination	11.244	1.694	6.609	1.134
Lumbar curve angle	46.738	3.451	41.023	5.516
LH/FSH	2.365	0.384	1.023	0.375

*LH/FSH* luteinizing hormone/follicle-stimulating hormone, *SD* standard deviation.

**Table 3:** Multiple pairwise comparison tests of the measured variables in the study and control groups.

Measured variables	*P*-value	95% Confidence interval for difference		Effect size (Cohen’s d)
		Lower bound	Upper bound	
Right pelvic inclination	0.000*	4.306	5.528	3.363
Left pelvic inclination	0.000*	4.034	5.236	3.217
Lumbar curve angle	0.000*	3.872	7.558	1.242
LH/FSH	0.000*	1.186	1.498	3.532

*Significant at alpha level < 0.05; *LH/FSH* luteinizing hormone/follicle-stimulating hormone.

### Correlation between scores of pelvic inclination and hormones (LH/FSH) of participants in the study

There was a strong positive correlation between the mean value of right pelvic inclination (*r* = 0.963, *p* = 0.01) and left pelvic inclination (*r* = 0.933, *p* = 0.01) and hormones (LH/FSH) of the participants who took part in the study.

### Correlation between scores of lumbar curve angle and hormones (LH/FSH) of participants in the study

There was a strong positive correlation (*r* = 0.784, *p* = 0.01) between and the mean value of the lumbar curve angle and the hormones (LH/FSH) of the participants who took part in the study.

## Discussion

Polycystic ovarian syndrome (PCOS) carries a significant long-term health risk that could cause hypertension, cancer, metabolic issues, cardiovascular problems, psychological effects, and reproductive challenges^2^. These conditions often manifest earlier in individuals with PCOS compared to their peers^2,25^. Those with PCOS and BMI over 25 face a greater risk of these long-term issues than those with a lower BMI^25^. Spinal posture, flexibility, and pelvic alignment are key components routinely evaluated in clinical settings during musculoskeletal assessments. They are vital for performing daily activities and a range of occupational and recreational tasks effectively^26^. Therefore, the purposes of this study were to determine if there were any biomechanical changes (pelvic inclination and lumbar curve angles) in females with PCOS and to verify the association between pelvic inclination, lumbar lordosis, and LH/FSH ratio in such cases.

The findings of the current study revealed that there were significant increases in the mean values of pelvic inclination angle and lumbar angle in group (A) compared with group (B). Additionally, there was a strong positive correlation between pelvic inclination (right and left), lumbar angle, and hormones (LH/FSH). The current findings should be explained in the context of widespread comorbidities linked with PCOS. One biomechanical explanation is that in women with PCOS, an imbalance of hormones, chronic low-grade inflammation, and less muscle mass and strength may cause their bodies to take actions that affect their balance and lumbopelvic alignment. The pelvis orientation and the lumbar lordosis curve can both have an impact on the overall sagittal balance^27^. The pelvis acts as a pivotal junction between the spine and hips, ensuring equilibrium during upright walking^28^. Hormonal changes linked to PCOS can also affect the tension and length of the pelvic floor muscles^9^. The coordinated action of the pelvic floor and core muscles is essential for movement, balance, stability, and flexibility^9,29^. Weakened pelvic floor muscles can disrupt this core synergy, leading to impaired muscle coordination^12^. Thus, in the current study, the increased lumbar lordosis and pelvic inclination in females with PCOS might occur because of this muscular imbalance.

Another physiological explanation for the increased pelvic tilting and lumber lordosis in group (A) compared with group (B) is the increased pain perception in patients with PCOS. Multiple pathologic factors related to PCOS may intensify pain perception, such as low-grade inflammation, oxidative stress, adipogenesis, and insulin resistance^30^. Research has indicated that pro-inflammatory factors, like cytokines and chemokines, are present at higher levels in the serum of PCOS patients compared to healthy individuals, highlighting the impact of inflammation on pain perception^31,32^. Additionally, it has been documented that inflammation is crucial in the genesis and persistence of various pain conditions within the peripheral and central nervous systems, and the link between chronic systemic inflammation and heightened pain sensitivity is deeply involved in the onset and continuation of chronic pain^33^. Therefore, the proinflammatory status may play a role in the pain experienced in females with PCOS.

Pain can influence lumbopelvic alignment, body function, and overall quality of life. Walicka-Cupryś et al. (2023) studied the impact of menstrual pain on lumbar spine mobility and postural alignment. The study found significant differences between the women who had menstrual pain and the control group in the angles of lumbar-sacral transition, thoracolumbar transition, lumbar lordosis, and thoracic kyphosis^34^. Karakus et al. found a significant positive correlation between pelvic mobility in the sagittal plane and the anterior lumbar angle in women with primary dysmenorrhea compared to asymptomatic women^35^. Additionally, Kim et al. observed an increase in the lordotic angle in women with dysmenorrhea^36^.

The metabolic explanation for the significant increases in lumbar lordosis and sagittal pelvic tilt angles observed in group (A) compared to group (B) may be caused by the impact of PCOS on muscle function. PCOS can affect muscular function through various metabolic and hormonal factors, including hyperandrogenism, obesity, insulin resistance, and levels of high-density lipoprotein cholesterol (HDL)^14^. Insulin, a primary muscle protein regulator, can enhance mitochondrial protein synthesis. Androgens may boost muscle strength or endurance and support the growth of lean muscle mass. Conversely, a reduction in HDL can impair muscle function by promoting the release of proinflammatory cytokines. Core strength and endurance, involving abdominal, paraspinal, gluteal, diaphragm, and pelvic floor muscles, are aspects of muscle function that PCOS can influence due to altered biochemical profiles. Changes in muscle function can reduce physical awareness and feedback from the body, potentially leading to abnormal body alignment^37^.

Doan et al. observed a reduction in aerobic capacity, muscle endurance, body awareness, and quality of life in normal-weight women with PCOS compared to controls with similar androgen levels and BMI profiles. Additionally, they identified a significant correlation between core endurance tests, HDL cholesterol, maximum oxygen consumption, and homeostatic model assessment for insulin resistance. These findings indicate that the detrimental metabolic profile associated with PCOS may impair physical function^14^.

Certain symptoms and health risks associated with PCOS can potentially diminish the musculoskeletal health. Patients with PCOS often have insulin resistance, hormone imbalances, chronic inflammation, low vitamin D levels, and inactive lifestyle. These are all symptoms of musculoskeletal disorders like osteosarcopenia, which may start to appear in mid-life. Osteosarcopenia combines both sarcopenia and osteopenia or osteoporosis. Having both sarcopenia and osteopenia or osteoporosis together is very dangerous because of the complicated way that endocrine and paracrine signals affect bone and muscle^15,38^. Kazemi et al. found that women with PCOS show early signs of osteosarcopenia, such as lower bone mineral density and lean body mass, even when age and obesity are considered. This suggests that insulin dysfunction may be a cause of muscle and bone loss in PCOS^15^.

The current study showed a strong positive correlation between pelvic inclination (Right and Left), lumbar angle, and hormones (LH/FSH). This means that an increase in the LH/FSH ratio is consistent with the increase in lumbopelvic misalignment. The findings of the current study are in line with those of Kazemi et al., who reported that women with PCOS had an increased LH/FSH ratio compared to controls^15^.

Another hazardous effect of PCOS that could affect the lumbopelvic alignment is its association with symptoms related to autoimmune diseases such as “joint pain,” “joint swelling,” and “pain in the heel” than the controls. Additionally, it was reported that women with PCOS exhibit a higher prevalence and earlier onset of osteoarthritis in both weight-bearing and non-weight-bearing joints compared to controls^16^. The incidence rates of hip, knee, and hand osteoarthritis are significantly higher in the PCOS group than in controls. This association persists even when excluding obese women, particularly for knee and hand osteoarthritis^16^. The biological reasons why PCOS raises the risk of osteoarthritis include excessive joint loading and the effects of metabolic dysregulation and systemic inflammation on joint tissue regeneration and remodeling^16^.

The treatment and evaluation of PCOS have become increasingly significant due to its chronic nature. In managing PCOS, assessing biomechanical parameters like the pelvic inclination angle and lumbar curve angle is crucial to prevent adverse outcomes. Postural changes, such as increased lumbar lordosis and pelvic tilting, stretch the abdominal muscles, weaken pelvic muscle strength and lower intra-abdominal pressure^39^, destabilize the lumbar spine and worsen the lumbar curvature. This increased load on the lumbar region can cause muscle fatigue and pain. Moreover, the lumbar intervertebral discs and facet joints may suffer from changes in lumbar curvature, leading to possible degeneration^40^. Exacerbated lumbar lordosis and anterior pelvic tilting can disrupt mechanical balance, potentially causing fatigue in lumbar back muscles^41,42^ and altering stress distribution in the lumbar spine^43^. These alterations may lead to instability in the posterior facet joints, increased shear stress, and organic changes like cartilage degeneration and spinal canal narrowing^44^. Therefore, the findings of the current study urge researchers and healthcare professionals to take into consideration the negative impact of PCOS on musculoskeletal health and the importance of implementing management strategies to prevent lumbopelvic misalignment in this patient group.

The findings of the current study are limited to the tested adult population and the selected range of BMI. Larger BMI wasn’t examined to avoid the considerable consequences for the obesity degrees on the measured parameters. The BMI only was used for comparing the anthropometric measurements between the control and patient groups in the present study. Future studies are recommended to measure the waist circumference in addition to BMI for assessing the abdominal obesity. In the current study, we measured only the lumbopelvic parameters. Future studies should be conducted to investigate the effect of PCOS on other biomechanical parameters such as thoracic and cervical parameters, musculoskeletal disorders and postural changes. The current study involved only nulliparous females with no obstetric history because pregnancy and difficult birth history can lead to permanent changes in pelvic and lumbar muscles in females. Hence, the obstetric history is a confounding factor that could affect the results of our study.

## Conclusion

Females with PCOS had greater pelvic tilting and exaggerated lumbar lordosis than controls. The LH/FSH ratio exhibited a strong correlation with both the lumbar curve angle and pelvic inclination in females with PCOS.

## Data Availability

The datasets generated and analyzed during the current study are not publicly available due to IP and confidentiality concerns that prevent their sharing. However, the corresponding author can provide them upon reasonable request.

## References

[1] Dapas, M. et al. Distinct subtypes of polycystic ovary syndrome with novel genetic associations: an unsupervised, phenotypic clustering analysis. *PLoS Med.* **17**, e1003132 (2020). 32574161 10.1371/journal.pmed.1003132 PMC7310679

[2] Teede, H. J. et al. Recommendations from the international evidence-based guideline for the assessment and management of polycystic ovary syndrome. *Clin. Endocrinol.* **89**, 251–268 (2018). 10.1111/cen.13795 PMC9052397 30024653

[3] De Leo, V. et al. Genetic, hormonal, and metabolic aspects of PCOS: an update. *Reprod. Biol. Endocrinol.* **14** (1), 1–7 (2016). 27423183 10.1186/s12958-016-0173-x PMC4947298

[4] Shorakae, S. et al. Inter-related effects of insulin resistance, hyperandrogenism, sympathetic dysfunction, and chronic inflammation in PCOS. *Clin. Endocrinol.* **89**, 628–633 (2018). 10.1111/cen.13808 29992612

[5] Parker, J. Understanding the pathogenesis of polycystic ovary syndrome: transgenerational evolutionary adaptation to lifestyle and the environment. *J. Australas. Coll. Nutr. Environ. Med.* **39** (4), 18–26 (2020).

[6] Rudnicka, E. et al. Chronic low-grade inflammation in the pathogenesis of PCOS. *Int. J. Mol. Sci.* **22** (7), 3789 (2021). 33917519 10.3390/ijms22073789 PMC8038770

[7] Crafa, A. et al. The burden of hormonal disorders: a worldwide overview with a particular look in Italy. *Front. Endocrinol.* **12**, 694325 (2021). 10.3389/fendo.2021.694325 PMC8242938 34220719

[8] Rostamtabar, M. et al. Pathophysiological roles of chronic low-grade inflammation mediators in polycystic ovary syndrome. *J. Cell. Physiol.* **236** (2), 824–838 (2021). 32617971 10.1002/jcp.29912

[9] Ptaszkowski, K. et al. Assessment of bioelectrical activity of pelvic floor muscles depending on the orientation of the pelvis in menopausal women with symptoms of stress urinary incontinence: continued observational study. *Eur. J. Phys. Rehab. Med.* **53**, 564–574 (2017). 10.23736/S1973-9087.17.04475-6 28145398

[10] Bureau, M. & Carlson, K. V. Pelvic organ prolapse: a primer for urologists. *Can. Urol. Assoc. J.* **11** (6 Suppl 2), S125 (2017). 28616110 10.5489/cuaj.4634 PMC5461143

[11] Saei Ghare Naz, M. et al. Polycystic ovary syndrome and pelvic floor dysfunction: a narrative review. *Res. Rep. Urol.* **12**, 179–185 (2020). 32440514 10.2147/RRU.S249611 PMC7213900

[12] Nipa, S. I., Sriboonreung, T., Paungmali, A. & Phongnarisorn, C. The effects of pelvic floor muscle exercise combined with core stability exercise on women with stress urinary incontinence following the treatment of nonspecific chronic low back pain. Adv. Urol.** 2022**, 2051374 (2022). 10.1155/2022/2051374 10.1155/2022/2051374 PMC9467742 36105867

[13] Bussey, M. D. et al. Is pelvic floor dysfunction associated with the development of transient low back pain during prolonged standing? A protocol. *Clin. Med. Insights: Women’s Health*. **12**, 1179562X19849603 (2019). 10.1177/1179562X19849603 PMC6537301 31205437

[14] Doğan, H. & Çaltekin, M. D. Does polycystic ovary syndrome with phenotype D affect cardiovascular endurance, core endurance, body awareness, and quality of life? A prospective, controlled study. *Turk. J. Obstet. Gynecol.* **18** (3), 203 (2021). 34580552 10.4274/tjod.galenos.2021.72547 PMC8480217

[15] Kazemi, M. et al. Osteosarcopenia in reproductive-aged women with polycystic ovary syndrome: a multicenter case–control study. *J. Clin. Endocrinol. Metab.* **105** (9), e3400–e3414 (2020). 32614948 10.1210/clinem/dgaa426 PMC7418445

[16] Kluzek, S. et al. Accelerated osteoarthritis in women with polycystic ovary syndrome: a prospective nationwide registry-based cohort study. *Arthritis Res. Ther.* **23** (1), 225 (2021). 34461982 10.1186/s13075-021-02604-w PMC8406767

[17] Teede, H. J. et al. Recommendations from the international evidence-based guideline for the assessment and management of polycystic ovary syndrome. *Hum. Reprod.* **33** (9), 1602–1618 (2018). 30052961 10.1093/humrep/dey256 PMC6112576

[18] Zhoolideh, P. et al. The relationship between static standing posture and common pelvic floor disorders. *Muscles Ligaments Tendons J.* **11** (1), 77–82 (2021).

[19] Reddy, R. A. et al. Role of sex steroid hormones in pelvic organ prolapse. *Menopause* **27** (8), 941–951 (2020). 32301895 10.1097/GME.0000000000001546

[20] Vardy, M. D. et al. The effects of hormone replacement on the biomechanical properties of the uterosacral and round ligaments in the monkey model. *Am. J. Obstet. Gynecol.* **192** (5), 1741–1751 (2005). 15902188 10.1016/j.ajog.2004.10.639

[21] Suits, W. H. Clinical measures of pelvic tilt in physical therapy. *Int. J. Sports Phys. Ther.* **16** (5), 1366 (2021). 34631258 10.26603/001c.27978 PMC8486407

[22] Vila-Casademunt, A. et al. The reliability of sagittal pelvic parameters: the effect of lumbosacral instrumentation and measurement experience. *Spine* **40** (4), E253–E258 (2015). 25494319 10.1097/BRS.0000000000000720

[23] Pourahmadi, M. et al. The reliability and concurrent validity of a new iPhone^®^ application for measuring active lumbar spine flexion and extension range of motion in patients with low back pain. *Physiother. Theory Pract.* (2021). 10.1080/09593985.2019.1616017 31081417

[24] Naqvi, S. Z. et al. Measurement of lumbosacral angle in normal radiographs: a cross-sectional study. *J. Liaquat Univ. Med. Health Sci.* **19** (04), 238–241 (2020).

[25] Dason, E. S. et al. Diagnosis and management of polycystic ovarian syndrome. *CMAJ* **196** (3), E85–E94 (2024). 38286488 10.1503/cmaj.231251 PMC10833093

[26] Qi, H. et al. Association of body mass index and central obesity with spino-pelvic alignment parameters in a Chinese population: a prospective study.* World Neurosurg.* (2024). 10.1016/j.wneu.2024.06.005 38857870

[27] Sharma, T. et al. Neuromechanical characterization of the abductor hallucis and its potential role in upright postural control. *Appl. Physiol. Nutr. Metab.* **49** (3), 293–305 (2023). 37913527 10.1139/apnm-2023-0226

[28] Vergari, C. et al. The relationship between spino-pelvic-hip mobility and quality of life before and after total hip arthroplasty. *Arch. Orthop. Trauma Surg.* **144** (3), 1379–1387 (2024). 37847287 10.1007/s00402-023-05094-4

[29] Son, S. M. Influence of obesity on postural stability in young adults. *Osong Public Health Res. Perspect.* **7** (6), 378–381 (2016). 28053843 10.1016/j.phrp.2016.10.001 PMC5194219

[30] Lu, K. T. et al. Evaluation of bodily pain associated with polycystic ovary syndrome: a review of health-related quality of life and potential risk factors. *Biomedicines* **10** (12), 3197 (2022). 36551953 10.3390/biomedicines10123197 PMC9776021

[31] Regidor, P. A. et al. PCOS: a chronic disease that fails to produce adequately specialized pro-resolving lipid mediators (SPMs). *Biomedicines* **10** (2), 456 (2022). 35203665 10.3390/biomedicines10020456 PMC8962413

[32] Aboeldalyl, S. et al. The role of chronic inflammation in polycystic ovarian syndrome—a systematic review and meta-analysis. *Int. J. Mol. Sci.* **22** (5), 2734 (2021). 33800490 10.3390/ijms22052734 PMC7962967

[33] Baral, P., Udit, S. & Chiu, I. M. Pain and immunity: implications for host defense. *Nat. Rev. Immunol.* **19** (7), 433–447 (2019). 30874629 10.1038/s41577-019-0147-2 PMC6700742

[34] Walicka-Cupryś, K. et al. Effect of lumbar spine mobility and postural alignment on menstrual pain in young women. *Int. J. Environ. Res. Public Health*. **20** (15), 6458 (2023). 37568998 10.3390/ijerph20156458 PMC10418796

[35] Karakus, A. et al. Lumbopelvic muscle endurance, morphology, alignment, and mobility in women with primary dysmenorrhea: A case–control study. *Clin. Biomech.* **92**, 105582 (2022). 10.1016/j.clinbiomech.2022.105582 35093799

[36] Kim, M. J., Baek, I. H. & Goo, B. O. The effect of lumbar-pelvic alignment and abdominal muscle thickness on primary dysmenorrhea. *J. Phys. Ther. Sci.* **28** (10), 2988–2990 (2016). 27821975 10.1589/jpts.28.2988 PMC5088166

[37] Tuttle, C. S., Thang, L. A. & Maier, A. B. Markers of inflammation and their association with muscle strength and mass: A systematic review and meta-analysis. *Aging Res. Rev.* **64**, 101185 (2020). 10.1016/j.arr.2020.101185 32992047

[38] Kazemi, M. et al. Comprehensive evaluation of type 2 diabetes and cardiovascular disease risk profiles in reproductive-age women with polycystic ovary syndrome: a large Canadian cohort. *J. Obstet. Gynaecol. Can.* **41** (10), 1453–1460 (2019). 30712903 10.1016/j.jogc.2018.11.026

[39] Baek, S., Park, H. W. & Kim, G. Associations between trunk muscle/fat composition, narrowing lumbar disc space, and low back pain in middle-aged farmers: a cross-sectional study. *Ann. Rehab. Med.* **46** (3), 122–132 (2022). 10.5535/arm.21201 PMC9263327 35793901

[40] Hawellek, T. et al. Microcalcification of lumbar spine intervertebral discs and facet joints is associated with cartilage degeneration but differs in prevalence and its relation to age. *J. Orthop. Res.* **35** (12), 2692–2699 (2017). 28467655 10.1002/jor.23591

[41] Kouyoumdjian, P. et al. Hip-spine relationship between the sagittal balance of the lumbo-pelvic-femoral complex and hip extension capacity: an EOS evaluation in a healthy Caucasian population. *Global Spine J.* **14**, 265–271 (2024). 35604878 10.1177/21925682221103831 PMC10676160

[42] Leong, C. H., Forsythe, C. & Bohling, Z. Posterior chain and core training improves pelvic posture, hamstrings-to-quadriceps ratio, and vertical jump performance. *J. Sports Med. Phys. Fit.* (2023). 10.23736/S0022-4707.23.15171-1 37800401

[43] Tan, L. X., Du, X. K., Tang, R. M., Rong, L. M. & Zhang, L. M. Effect of spinal-pelvic sagittal balance on the clinical outcomes after lumbar fusion surgery. *BMC Surg.* **23** (1), 334 (2023). 37914985 10.1186/s12893-023-02240-y PMC10621172

[44] Cornaz, F. et al. Intervertebral disc degeneration relates to Biomechanical changes of spinal ligaments. *Spine J.* **21** (8), 1399–1407 (2021). 33901629 10.1016/j.spinee.2021.04.016

